# Veratramine ameliorates pain symptoms in rats with diabetic peripheral neuropathy by inhibiting activation of the SIGMAR1-NMDAR pathway

**DOI:** 10.1080/13880209.2022.2136207

**Published:** 2022-11-14

**Authors:** Yu Zhang, Guangyao Ye, Yuebo Chen, Chaoxu Sheng, Jianlin Wang, Lingsi Kong, Liyong Yuan, Chunyan Lin

**Affiliations:** Department of Anesthesiology, Ningbo No.6 Hospital, Ningbo, P. R. China

**Keywords:** Neuropathic pain, high-fat diet, mechanical withdrawal threshold, coimmunoprecipitation, sciatic nerve pathological injury

## Abstract

**Context:**

Veratramine may have a potential therapeutic effect for diabetic peripheral neuropathy (DPN).

**Objective:**

To evaluate whether veratramine ameliorates neuropathic pain in a rat diabetic model.

**Materials and methods:**

Sprague–Dawley rats were used for a diabetic model induced by a streptozotocin + high-fat diet. Two months after the induction of the diabetic model, the rats with DPN were screened according to the mechanical pain threshold. The rats with DPN were divided into a model group (n = 12) and a treated group (n = 12). Rats with diabetes, but without peripheral neuropathy, were used in the vehicle group (n = 9). The treatment group received 50 μg/kg veratramine via the tail vein once a day for 4 weeks. During modelling and treatment, rats in all three groups were fed a high-fat diet.

**Results:**

The mechanical withdrawal threshold increased from 7.5 ± 1.9 N to 17.9 ± 2.6 N in DPN rats treated with veratramine. The tolerance time of the treated group to hot and cold ectopic pain increased from 11.8 ± 4.2 s and 3.4 ± 0.8 s to 20.4 ± 4.1 s and 5.9 ± 1.7 s, respectively. Veratramine effectively alleviated L4-L5 spinal cord and sciatic nerve pathological injury. Veratramine inhibited the expression of SIGMAR1 and the phosphorylation of the N-methyl-d-aspartate receptor (NMDAR) Ser896 site in spinal cord tissue, as well as inhibited the formation of SIGMAR1-NMDAR and NMDAR-CaMKII complexes.

**Discussion and conclusions:**

Veratramine may alleviate the occurrence of pain symptoms in rats with DPN by inhibiting activation of the SIGMAR1-NMDAR pathway.

## Introduction

Diabetes is one of the fastest-growing health challenges of the 21st century, with the number of adults with the condition more than tripling in the past 20 years (Tönnies et al. 2021). Sustained high blood sugar levels can lead to severe diseases, and diabetic peripheral neuropathy (DPN) is the most common complication, occurring in approximately 50% of patients with diabetes (Hicks and Selvin 2019). Common symptoms of DPN include burning, tingling, and pain beginning in the distal extremities and can progress to more extreme neuropathic symptoms in approximately 10–30% of affected patients (Sloan et al. 2018).

At present, tricyclic drugs (TCA), serotonin-norepinephrine reuptake inhibitors (SNRIs), or anticonvulsants (gabapentin or pregabalin) are used as first-line drugs for DPN treatment, followed by opioids and local therapy (Spallone 2012). Although these drugs have been the subject of formal clinical trials, they do not prevent the progression of the disease, and long-term use can bring a variety of toxic side effects. For example, opioids can cause nausea, itching, dizziness, inhibition of the pituitary axis, immunological changes, and the possibility of dependence and abuse. Anticonvulsants can cause mood disorders, peripheral edema, and even seizures (Javed et al. 2015). Therefore, it is urgent to better understand the pathogenesis of DPN and develop more effective drugs to treat it.

*Veratrum nigrum* Linn. (Melanthiaceae), a perennial herb, has a rich clinical history in Europe, Asia, and North America (Chandler and McDougal 2014). Dadix Veratri, mainly derived from *Veratrum nigrum*, is one of the primary components of the formulation of the traditional Chinese medicine of ‘Yunnan Baiyao’ and the main drug of the protected traditional Chinese medicine ‘Yizhi Zhitong Pill’. Veratramine is one of the main extracts of Dadix Veratri (Lyu et al. 2016). Veratramine is a known natural steroid alkaloid found in various Liliaceae plants (Li et al. 2019). Due to the vital role of Dadix Veratri in analgesia, we hypothesised that veratramine might also play an essential role in analgesia. Previous studies by Li et al. (2016, 2019) showed that veratramine has strong anti-inflammatory and analgesic effects. Although the authors demonstrated that the main analgesic ingredient of *veratrum* was veratramine (Li et al. 2016, 2019), they did not provide an in-depth discussion on the specific analgesic mechanism of veratramine.

Therefore, in this study, we first examined the efficacy of veratramine in the treatment of DPN and then combined it with small molecule simulation docking technology to predict the potential target protein between veratramine and DPN. The results suggest that sigma nonopioid intracellular receptor 1 (SIGMAR1) may be an important target of veratramine. It has been previously shown that inhibition of SIGMAR1 can inhibit microglial activation and reduce mechanical hypersensitivity, thus relieving pain in chronic osteoarthritis (Carcolé et al. 2019). In addition, a SIGMAR1 antagonist has been shown to alleviate nerve pain in mice with chronic sciatic nerve contraction injury (Rodríguez-Muñoz et al. 2021). Here, we combine these concepts in experiments designed to investigate the specific mechanism of the alleviation of DPN by veratramine.

## Materials and methods

### Animals

Forty male 8-week-old Sprague Dawley rats (weighing 260–300 g) were purchased from Shanghai Jiesijie Experimental Animal Co., Ltd. Normal drinking water and rat feed were provided, constant humidity and temperature were maintained, and a 12 h circadian cycle was provided for adaptive feeding for one week. The animal experiment protocol in this study was approved by the Animal Ethics Committee of Ningbo University (No. 10431). According to animal welfare requirements (Jar 2014), the rats that could not continue the experiment were euthanized.

### DPN model

The rat diabetes model was constructed after appropriate adjustment as previously described (Guo et al. 2018; Tang et al. 2020). After 1 week of adaptive feeding, rats were induced to consume a high-fat diet (CAT No. XTHF45, Jiangsu Xietong Pharmaceutical Biological Engineering Co. LTD) for 1 month. After one month, 20 mg/mL streptozotocin (STZ) was prepared with 0.1 M sodium citrate-citric acid buffer (pH 4.5), and the rats were intraperitoneally injected with 60 mg/kg STZ (single injection). The symptoms of the rats were observed after STZ injection, and tail vein blood was collected on the fourth day to detect blood glucose. Diabetes was considered to be successfully modelled when the fasting blood glucose (FBG) values were >13.8 mmol/L, and the rats that did not develop diabetes were excluded. After 2 months of diabetes induction, pain threshold tests were performed on the successfully modelled diabetic rats. Diabetic rats with DPN were screened according to the mechanical pain threshold, and rats that demonstrated successfully induced DPN were divided into two groups. The model group (model, *n* = 12) was given a high-fat diet (60% basic diet, 20% sucrose, 15% oil, and 5% cholesterol), and the treated group (treated, *n* = 12) was injected with 50 μg/kg veratramine (MedChemExpress Reagents Inc. Purity: 99.61%. 0.9% NaCl containing 5% PEG400 for dissolution, 40 μg/mL for injection concentration) into the tail vein and given a high-fat diet at the same time (Lyu et al. 2016), 3 times/week for 4 weeks. Rats with diabetes but without peripheral neuropathy were used as the vehicle group (*n* = 9) and also fed a high-fat diet. At the end of the experiment, cold and heat pain and mechanical hyperalgesia levels were assessed, and FBG levels were measured. The rats were anaesthetized with 35 mg/kg phenobarbital sodium, and the pancreas, sciatic nerve, and L4-L5 spinal cord tissues were rapidly removed and fixed in neutral formaldehyde for pathological examination, including hematoxylin-eosin (H&E) staining and immunohistochemistry (IHC). Finally, 100 mg spinal cords were fixed with liquid nitrogen and stored at −80 °C.

### Determination of blood glucose

Fasting blood glucose values were measured using a ONE-TOUCH (UltraVue, China) portable glucometer after 8 h of food deprivation in rats (8:00 a.m–10:00 a.m.). Blood samples were collected by cutting the tail at the tip of the rat’s tail, and a drop of blood was placed on a glucometer with a 25-gauge glucometer test strip (ONETOUCH Ultra, China) for blood glucose determination. The glucose oxidase method was used for detection.

### Mechanical hyperalgesia test

A pressure needle was used to measure the mechanical paw withdrawal reflex threshold before grouping and at the end of treatment in each group to evaluate mechanical pain thresholds in rats (mechanical withdrawal threshold (MWT)). Before starting the experiment, the rats were placed in the observation box to allow them to adapt to the environment of the observation box for 30 min. For MWT determination, the rats to be tested were placed in a box, and after the rats were acclimated, the experimenter held a pressure detection needle connected with a small animal physiological monitor (Medlab, Nanjing Calvin Instrument Co., LTD., China) and vertically stimulated the hind limbs of the rats through wire mesh, and a small animal physiological monitor detected the pressure value of the needle. The rapid foot lift reaction in rats during stimulation or when the pressure detection needle was removed was recorded as a positive reaction, and it did not include the foot lift reaction caused by the animal’s body movement in the positive category. The test was performed once every 30 s, 5 times in a row, and the average pressure value of the pressure test was used as the lifting threshold. The maximum detection limit was 25 N (Ye et al. 2021).

### Thermal hyperalgesia assay

This assay was performed as previously described in Ye et al (2021). At the end of administration in each group, the thermal paw withdrawal latency (TWL) was measured to evaluate thermal hypersensitivity in rats. The rats were placed on a radiative heat pain detector. After standing for 10 min, the left and right heels of the rats were concentrated and irradiated with a Hargreaves radiant heat device with the same intensity (infrared setting 80, 7371, Ugo Basile, Comerio, Italy), and the time when the intense light irradiated the rats’ feet was recorded, providing the TWL. Measurements were taken at 5 min intervals, and the average of the three measurements was taken as the TWL. The upper limit of TWL was set at 25 s. TWL in the right foot was compared between rats in each group.

### Cold allodynia test

Cold allodynia experiments were performed as previously described in Lee et al. (2014). Cold allodynia sensitivity was assessed by stimulating the rat tail with cold water (4 °C). Rats were allowed to habituate to the scaffold before starting the behavioural test. The rat’s tail was dropped and placed in cold water, and the time until the rat’s tail began to swing was recorded as the cold allodynia latency with a cut-off time of 20 s. The experiment was repeated five times at 5 min intervals. Shorter cold allodynia latencies were considered to be more severe cold allodynia.

### H&E staining

Paraffin tissue sections of the pancreas, sciatic nerve, and spinal cord were prepared from rats in each group. Xylene was used to deparaffinize, followed by rehydration with graded concentrations of alcohol and then staining with haematoxylin and eosin. The sections were then dehydrated with conventional alcohol, cleared with xylene, mounted with neutral gum, and observed and photographed under a light microscope (DM500, Leica, Germany).

### Bioinformatics analysis

Genes related to diabetes and peripheral neuropathy were searched through the GeneCard website. Then, the SMILE number of veratramine was used to predict target proteins of veratramine in SEA (https://sea.bkslab.org), SwissTarget (http://www. swisstargetprediction.ch/), and MolTarPred (http://moultarpred.marseille.inserm.fr/). Target proteins were identified by intersection screening of genes associated with DPN genes. The 3D structure of the compound veratramine was downloaded from the PubChem database (Fig. https://pubchem.ncbi.nlm.nih.gov/). The 3D structures of SHH (PDB No. 6OEV) and SIGMAR1 (PDB No. 5HK2) were obtained from the RCSB Protein Data Bank (http://www.rcsb.org/). PyMOL software was used to extract the protein structure to remove water molecules and ligands, and AutoDock software was used to save them in PDBQT format. Molecular docking studies were performed using AutoDock Vina 1.1.2. According to the position of the protein ligand, the conformation with the largest absolute value of docking binding energy was selected for docking binding energy mode analysis, and the visualisation function of PyMOL molecular simulation software was used to draw the results.

### Immunohistochemistry

Spinal cord tissue sections were rehydrated in xylene and graded ethanol solutions. Antigen retrieval was performed in an autoclave at 121 °C for 20 min in citric acid buffer (pH = 6). Endogenous enzyme inactivation was performed using 3% H_2_O_2_. Incubation with rabbit anti-SIGMAR1 (1:200, cat no. 15168-1-AP, Proteintech) was performed overnight at 4 °C. After washing off the primary antibody, the cells were incubated with goat anti-rabbit IgG-HRP secondary antibody (1:200, cat no. AS014, ABclonal) for 1 h at room temperature, then visualised by DAB staining and counterstained with haematoxylin followed by alcohol dehydration, and finally cleared with xylene, mounted with neutral gum, and examined by light microscopy (DM500, Leica). Image-ProPlus 6.0 software was used to quantify the IHC results.

### qPCR

Total RNA was extracted from L4-L5 spinal cord tissue by the TRIzol method (Rio et al. 2010). mRNA was reverse transcribed into cDNA using the first Strand cDNA Synthesis Kit (gDNA Purge, Novoprotein, China). Primers were designed through NCBI, and the sequences were as follows: SHH-F: CGG CTG ATG ACT CAG AGG TG, SHH-R: TCC ACT GCT CGA CCC TCA TA. SIGMAR1-F: CAT GGC CAT TCG GGA CGA TA, R: GGA AGT CCT GGG TGC TGA AA. GAPDH-F: AGA CAG CCG CAT CTT CTT GT, R: CGA TAC GGC CAA ATC CGT TC. qPCR was performed using a SYBR qPCR Super Mix Plus kit (Novoprotein, China). The reaction mixture contained 20 ng cDNA, 1.5 mmol of each primer and 2.5 mM MgCl_2_ in a 20 μL system, and qPCR was performed on a 7500 Fast Real-Time PCR System (Applied Biosystems; Thermo Fisher Scientific, Inc.). Cycling conditions were: denaturation at 95 °C for 2 min; denaturation at 95 °C for 10 s and annealing at 60 °C for 10 s, 40 cycles total; and extension at 72 °C for 30 s. The 2^-ΔΔCt^ method was used to calculate the relative expression of genes (Livak and Schmittgen 2001).

### Western blotting

The protein expression levels of SIGMAR1, NMDAR, and p-NMDAR (Ser896) were detected by Western blotting (WB). L_4_-L_5_ spinal cord tissues were collected from rats. Then, RIPA lysis buffer and phosphatase inhibitors were added to the tissue, and the tissue was homogenised by a tissue fragmentation instrument (Qiagen, Germany) on ice. The samples were centrifuged at 10,000*g* at 4 °C for 15 min, and the supernatant was collected. The homogenate was then denatured at 100 °C for 5 min. The denatured protein was added to a 12.5% SDS–PAGE gel, and electrophoresis was terminated by running out the separation gel until bromophenol blue at 90 V. The protein was then transferred to PVDF (Millipore, Germany). Next, 3% BSA was used for blocking overnight at 4 °C. After blocking was completed, the membranes were washed in TBST three times, and rabbit anti-rat SIGMAR1 (1:1000, cat no. 15168-1-AP, Proteintech), NMDAR (1:1000, cat no. 5704S, Cell Signaling Technology), p-NMDAR (Ser896, cat no. ARG51606, Arigobio), or GAPDH (1:1000, 2118, CST) were added and incubated at room temperature for 2 h. After washing with TBST, goat anti-rabbit IgG-HRP secondary antibody (1:2000) was added and incubated at room temperature for 2 h. ECL chemiluminescence agent (Sharebio, China) was used for luminescence, and imaging was performed using the UVP Imaging System (ChemiDoc-It Imaging System, USA). Relative protein expression was calculated using ImageJ software using GAPDH as an internal reference protein.

### Coimmunoprecipitation

Interactions between SIGMAR1, NMDAR, and CaMKII were detected by coimmunoprecipitation (Co-IP). First, the protein was extracted with RIPA lysis buffer, and the protein lysate was pre-treated with Protein A/G agarose beads at 4 °C for 1 h to remove antibodies from the lysate. The lysate was then centrifuged at 12,000*g* for 10 min at 4 °C, and the supernatant was transferred to a new 1.5 mL EP tube. SIGMAR1 antibody (1:50) or NMDAR antibody (1:50) was mixed with the supernatant and incubated overnight at 4 °C to conjugate the antigen to the antibody. Protein A/G agarose beads were added and incubated at 4 °C for 6 h to allow immune complexes to bind to Protein A/G agarose beads (Santa Cruz, USA), and the samples were then centrifuged at 12,000*g* for 10 min at 4 °C and the supernatant was aspirated. Next, fresh lysates were added and centrifuged at 12,000*g* for 10 min, three times. Then, 160 μL of fresh lysates and 40 μL of loading buffer were added and heated for 5 min at 100 °C. The enriched proteins were then used for WB detection with NMDAR and CaMKII (1:1000, cat no. Ab134041, Abcam). GAPDH protein was used as an internal reference protein for normalisation. The results were quantified using ImageJ.

### Statistical analysis

Statistical analysis. All data from the current study are presented as the mean ± standard deviation based on three repetitions. All results were calculated using GraphPad Prism Software (version Prism 8; GraphPad Software, Inc.). A Student’s *t*-test was used for intergroup comparisons. Differences were analysed by one‑way ANOVA followed by Tukey’s *post hoc* test. *p* < 0.05 was considered to indicate a statistically significant difference.

## Results

### Veratramine alleviates pain in rats with DPN

**Figure 1.**
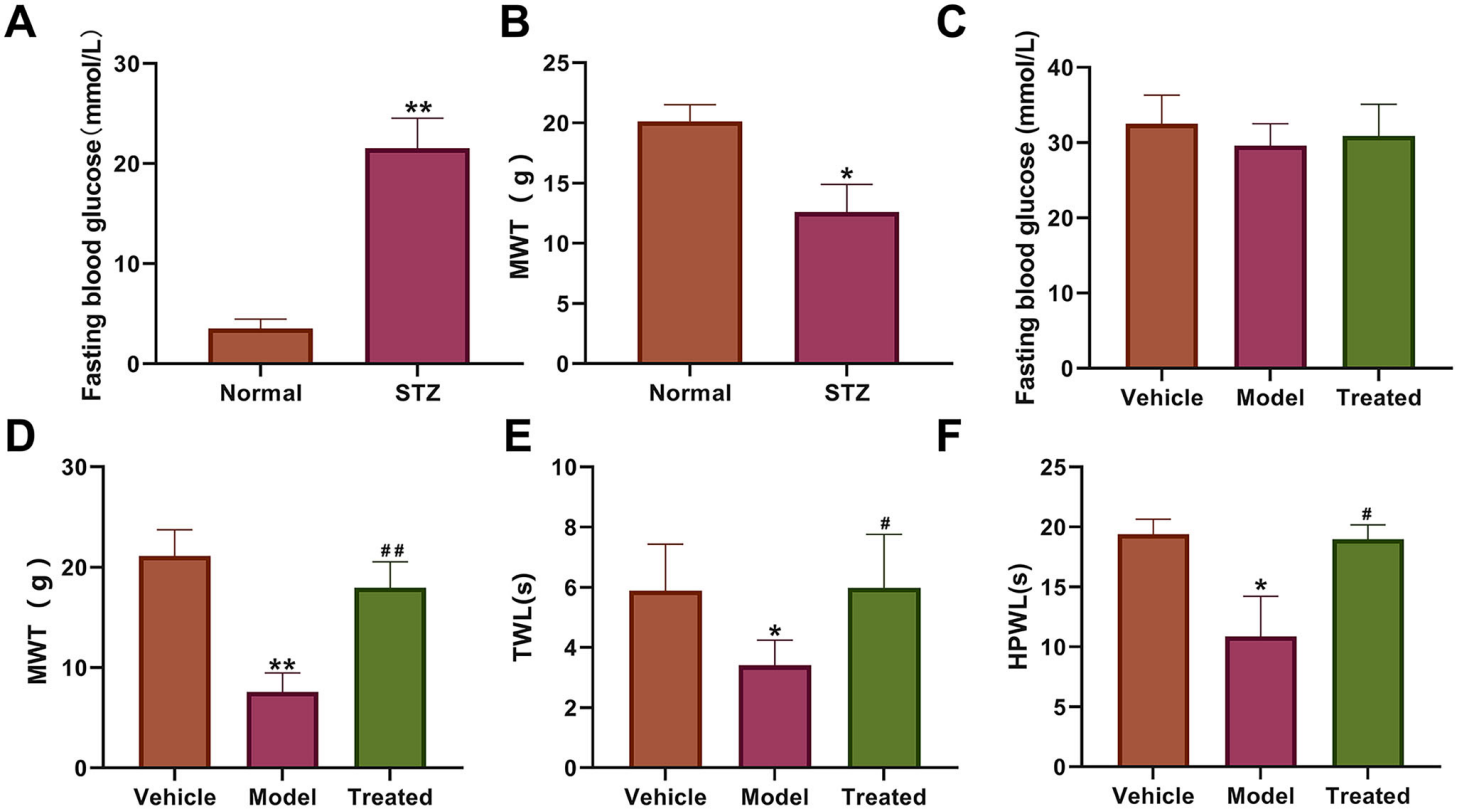
Effect of veratramine on pain parameters of diabetic peripheral neuropathy. (A) Fasting blood glucose values in rats; (B) Mechanical withdrawal threshold (MWT) in rats before veratramine administration; (C) FBG detection results of each group at the end of the experiment. (D) MWT in rats after veratramine administration; (E) Plantar thermal paw withdrawal latency in rats after veratramine administration; (F) Hind paw cold allodynia. **p < 0.01, *p < 0.05, vs. normal or vehicle group, ^##^*p* < 0.01, ^#^*p* < 0.05, treated group vs. model group.

To study the effects of veratramine on DPN in rats, a diabetic model was used. The results of one-touch detection of FBG in rats showed that the fasting blood glucose in the STZ group (21.54 ± 3.01 mmol/L) was significantly higher than that in the normal group (3.51 ± 0.94 mmol/L; *p* < 0.01, Figure 1(A)). The results of MWT detection in rats showed that the MWT was 20.13 ± 1.39 g in the normal group and 12.6 ± 2.68 g in the STZ group, which was significantly lower (*p* < 0.05, Figure 1(B)). The above results indicate the successful establishment of a DPN model in rats. At the end of the experiment, FBG detection data showed that the vehicle, model, and treated groups were 32.5 ± 3.8, 29.6 ± 2.9, and 30.9 ± 4.2 mmol/L, respectively, and the differences among the three groups were not statistically significant (*p* > 0.05, Figure 1(C)). The results of MWT detection in rats at the end of administration indicated that veratramine can effectively increase the MWT in diabetic rats with peripheral neuropathy. Veratramine increased the pain threshold to 17.96 ± 2.57 g compared with 7.57 ± 1.87 g in the model group, a highly significant difference between the two groups (*p* < 0.01, Figure 1(D)). The latency time of plantar heat radiation paw withdrawal in rats and the response time to heat allodynia in the model group were significantly shorter than those in the vehicle and treated groups (*p* < 0.05, Figure 1(E)), while there was no significant difference between the vehicle and treated groups (*p* > 0.05). The maximum endurance time of the tail to cold allodynia in the model group was 10.86 ± 3.36 s, and was increased to 18.97 ± 1.20 s after veratramine treatment, with a significant difference between the two groups (*p* < 0.05, Figure 1(F)). However, there was no significant difference between the vehicle and treated groups (*p* > 0.05).

### Veratramine alleviates spinal cord and sciatic nerve injury

**Figure 2.**
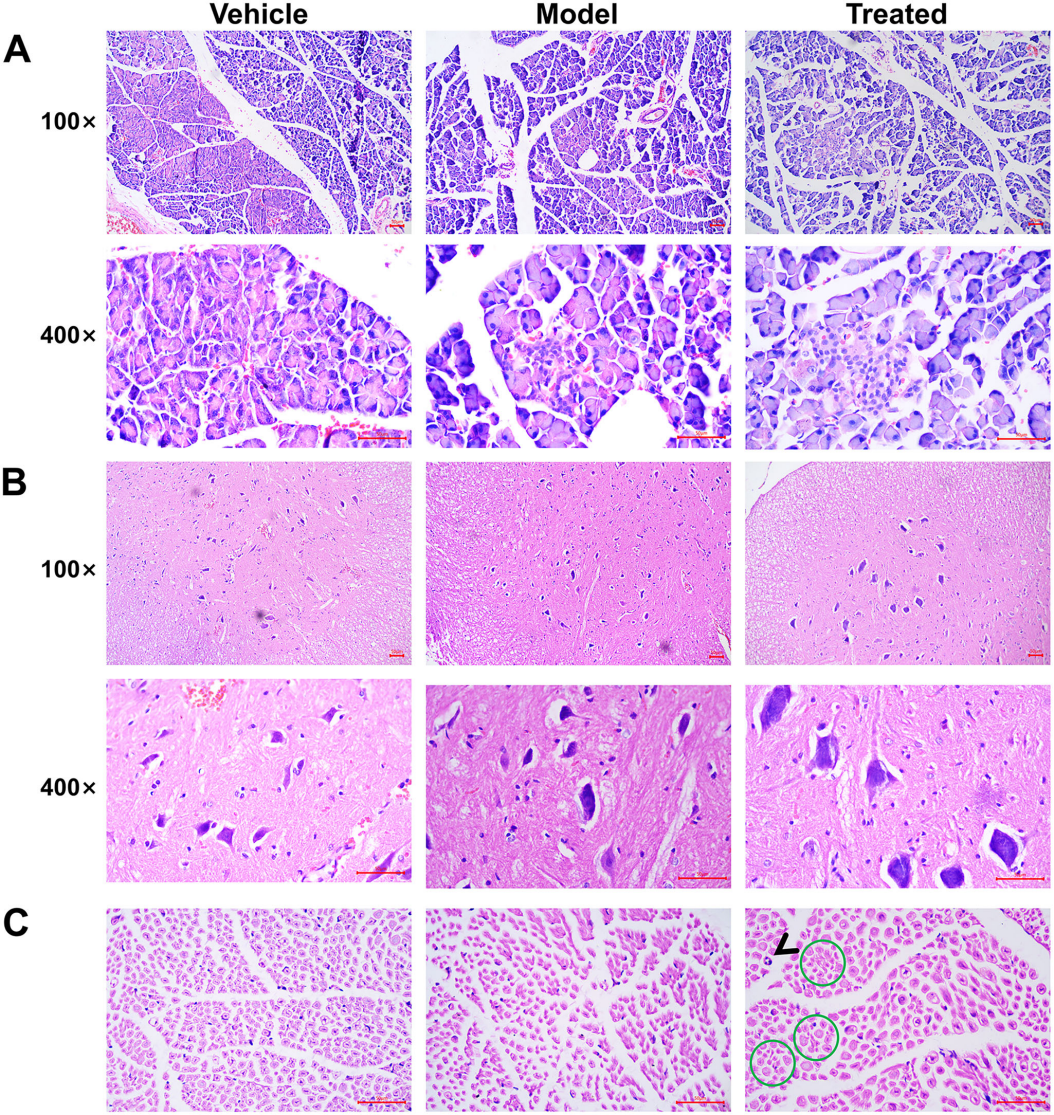
Pathological changes of peripheral neuropathy in aged diabetic rats treated with veratramine. **(**A) H&E photomicrographs of spinal cord tissue; (B) H&E photomicrographs of sciatic nerve. The arrows in the figure show that nerve cells show mitoses, and green circles represent nerve fibre enlargement or atrophy. The scale bar is 50 μm.

The results of H&E staining of spinal cord tissue showed that in the spinal cord of the model group, a large number of neuronal cells were atrophic, and the cell nuclei were pyknotic (Figure 2(A)). The expansion of neurons was observed in the spinal cord of the treated group, and the dendritic structure of neuronal cells was evident. H&E staining of the sciatic nerve showed partial degeneration of the axons and myelin sheaths of the sciatic nerve at the site of the green circle, and the black arrows indicate the division of myelin nuclei (Figure 2(B)). In the model group, a significant decrease in the number of large nerve fibres was observed in the sciatic nerve, and some small fibres nerve axons also showed degeneration.

### Veratramine regulates SIGMAR1 gene and protein expression

**Figure 3.**
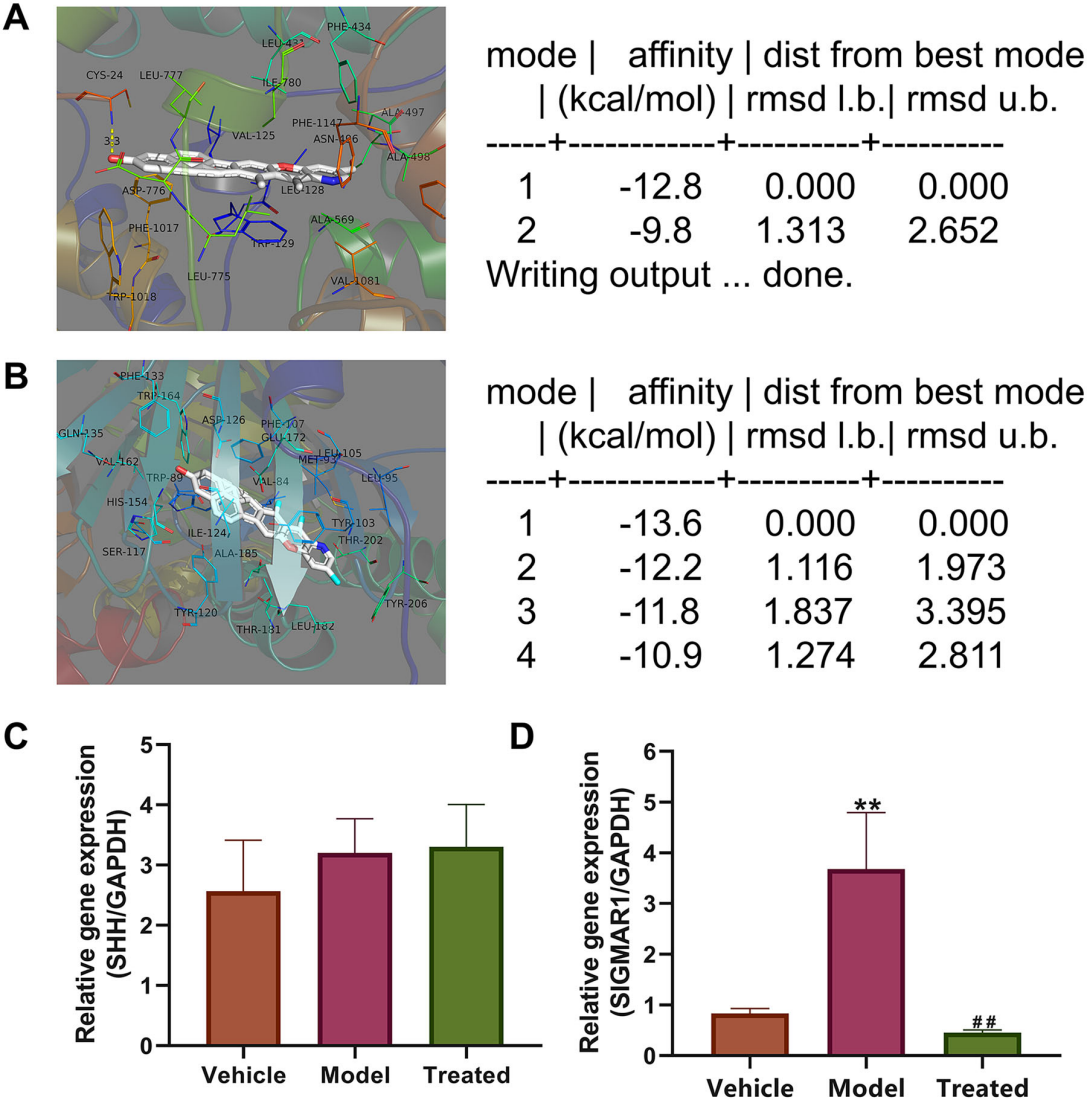
Bioinformatics screening of veratramine target proteins. (A) Veratramine was visualised by docking with SHH protein, and its docking binding energy was –12.8 kcal/mol; (B) Veratramine was visualised by docking with SIGMAR1 protein, and its docking binding energy was –13.6 kcal/mol; (C) qPCR was used to detect SHH gene expression levels in the spinal cord tissues of rats in each group; (D) The spinal cord tissues of rats in each group were examined for SIGMAR1 gene expression levels by qPCR. **p < 0.01, vs. vehicle group, ^##^*p* < 0.01, vs. model group.

**Figure 4.**
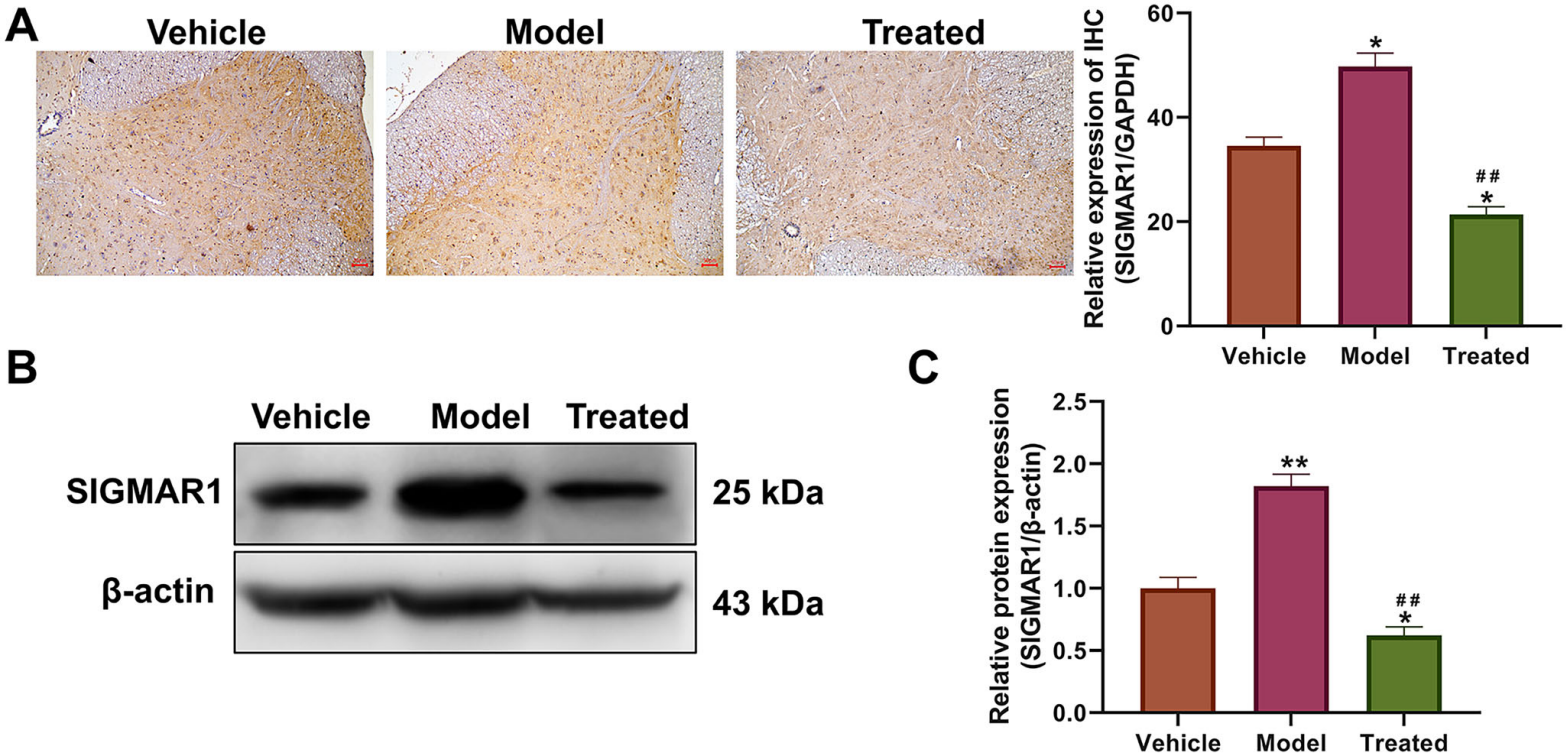
Effect of veratramine on SIGMAR1 protein expression in spinal cord tissue of diabetic rats. (A) Immunohistochemistry (IHC) was used to stain and localise SIGMAR1 protein in rat spinal cord tissue, and the figure on the right is a statistical quantification figure of IHC results. (B) WB was used to detect the protein expression level of SIGMAR1. (C) Statistical quantification results of grey values of WB bands. **p < 0.01, *p < 0.01, vs. vehicle group; ^##^*p* < 0.01, vs. model group. The scale bar is 50 μm.

Molecular docking was used to predict the interaction between the protein and drug. The binding energy of veratramine with the SHH protein was −12.8 kcal/mol (Figure 3(A)). The results showed docking of veratramine with SIGMAR1, with a binding energy of −3.6 kcal/mol (Figure 3(B)). The docking results showed that veratramine interacted with the above two proteins. The expression levels of the SHH and SIGMAR1 genes in spinal cord tissues were measured by qPCR (Figure 3(C,D)), and the SHH gene level did not change significantly in the spinal cord of rats in each group (*p* > 0.05). However, the gene expression level of SIGMAR1 was significantly upregulated in the model, while SIGMAR1 was significantly decreased after treatment with veratramine, a result that was significantly different from the model (*p* < 0.01) but not from the vehicle group (*p* > 0.05). Therefore, we used SIGMAR1 as a target gene for veratramine for subsequent experiments. IHC experiments showed that the SIGMAR1 expression level in the spinal cord of the model group was significantly higher than that of the vehicle group (*p* < 0.05; Figure 4(A)). From the localisation, it can be seen that SIGMAR1 expression is strongly positive in the grey matter and anterior and posterior horns of the spinal cord and is widely distributed in the spinal cord. After treatment with veratramine, the expression level of SIGMAR1 in the spinal cord was significantly inhibited compared with that in the vehicle and model groups (*p* < 0.05). SIGMAR1 protein expression levels in spinal cord tissues were also detected by WB (Figure 4(B,C)), and the results showed that SIGMAR1 expression levels in spinal cord tissues after veratramine treatment were significantly reduced and were even lower than those in the vehicle group (*p* < 0.05).

### Veratramine may influence neuronal pain regulation by modulating SIGMAR1 and its downstream pathways

**Figure 5.**
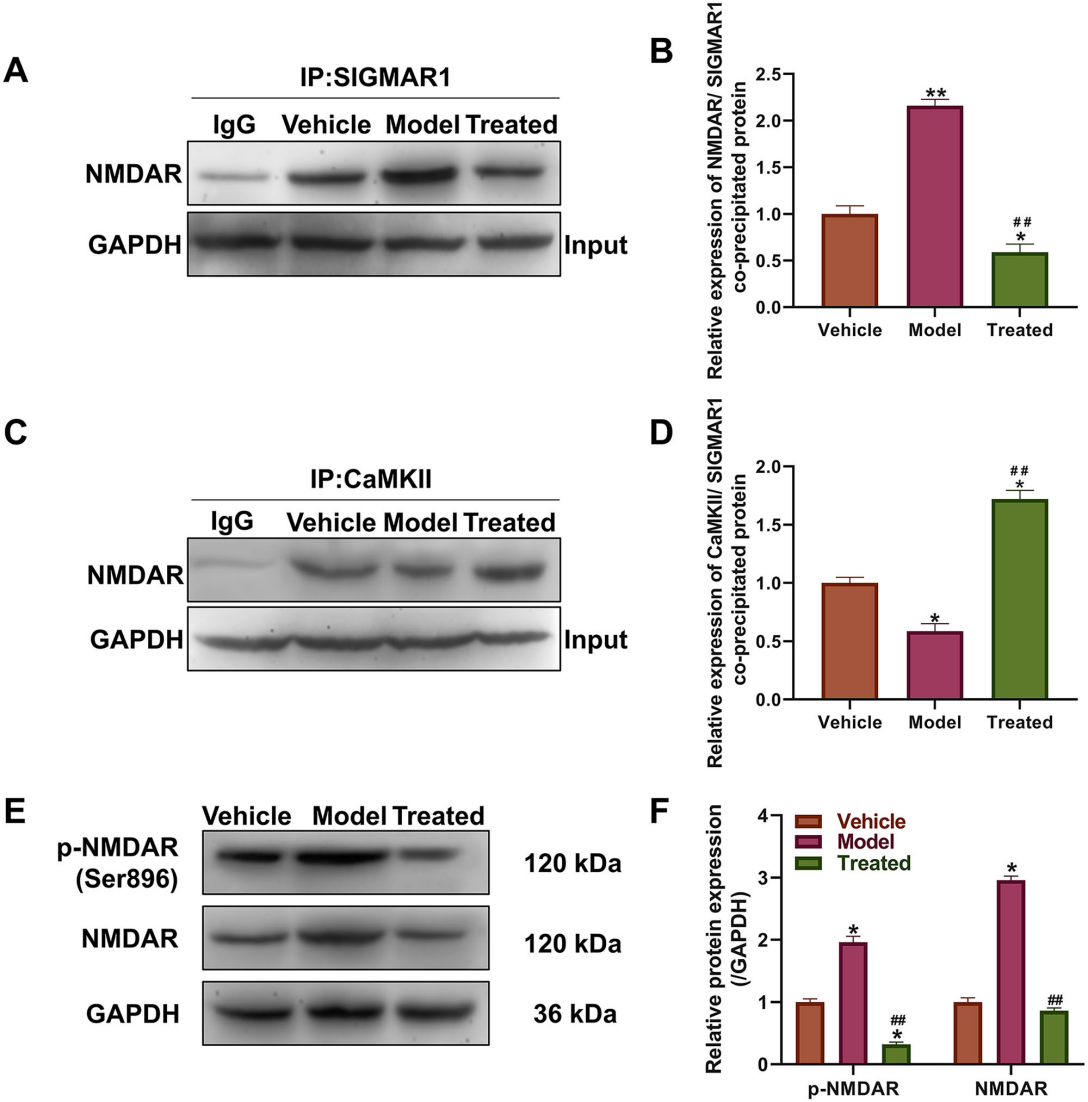
Effect of veratramine on SIGMAR1 and its downstream regulatory proteins. (A) Coimmunoprecipitation (Co-IP) results of SIGMAR1 with NMDAR protein. (B) Statistical quantification map of band grey values in result A; (C) Co-IP results of CaMKII with NMDAR protein. (D) Statistical quantification map of band grey values in results C; (E) WB was used to detect the expression levels of NMDAR and its phosphorylated proteins. (F) Statistical quantification plot of grey values of WB bands. **p < 0.01, *p < 0.01, vs. vehicle group; ^##^*p* < 0.01, vs. model group.

**Figure 6.**
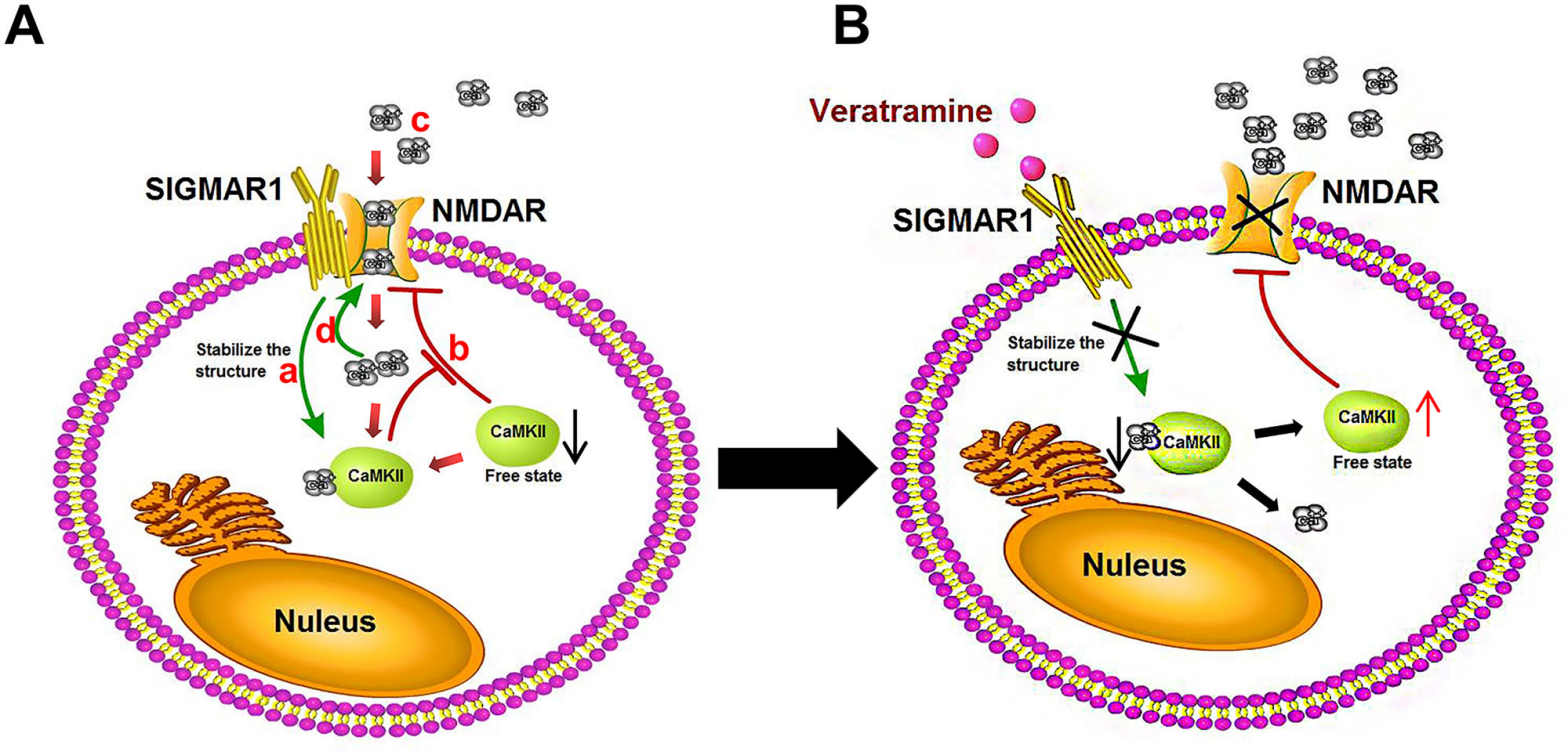
Mechanism of action of veratramine on the SIGMAR1-NMDAR pathway. A mechanistic diagram of the SIGMAR1-NMDAR pathway in the pathologically induced DPN state. (A) SIGMAR1 can stabilise the intracellular Ca^2+^-CAMKII structure. (B) The stable Ca^2+^-CaMKII complex structure leads to decreased CaMKII in the free state. The inhibitory effect of CaMKII protein on NMDAR is weakened, and the activity of NMDAR increases. (C) After NMDAR activity is increased, extracellular Ca^2+^ flows into the cell. (D) Increased intracellular Ca^2+^ concentration consolidates the SIGMAR1-NMDAR complex structure and further enhances NMDAR activity. (B) Effects of veratramine treatment on the SIGMAR1-NMDAR pathway: After SIGMAR1 activity is inhibited by veratramine, the stability of the Ca^2+^-CaMKII complex structure is decreased, and the free CaMKII protein is increased, thus inhibiting NMDAR activity. When NMDAR activity is decreased, Ca^2+^ inflow into cells decreases, and the stability of the SIGMAR1-NMDAR complex structure decreases, further weakening NMDAR protein activity.

Based on the results of small molecule simulation docking and western blot analysis, and considering the important role SIGMAR1 plays in pain relief, we further investigated its downstream influence pathway through a Co-IP experiment. The Co-IP experiment with SIGMAR1 and NMDAR protein showed that the protein level of SIGMAR1 and NMDAR protein coprecipitation was significantly higher than that of the vehicle and treated groups (*p* < 0.01, Figure 5(A,B)). After veratramine treatment, the coprecipitated protein decreased significantly (*p* < 0.01). Co-IP experiments were performed between CaMKII and NMDAR proteins (Figure 5(C,D)), and the results showed that the binding level of model CaMKII protein to NMDAR protein was significantly decreased compared with the vehicle group (*p* < 0.05), and the protein binding level was significantly increased in the treated group (*p* < 0.05). NMDAR and its phosphorylation levels were also examined by WB (Figure 5(E,F)). The results showed that the expression level of NMDAR protein was upregulated and that the phosphorylation of Ser896 was also activated in the model group compared with the vehicle group, and the phosphorylation level of NMDAR was significantly inhibited in the treated group (*p* < 0.05). The mechanism of action of veratramine on the SIGMAR1-NMDAR pathway is shown in Figure 6.

## Discussion

DPN is a complex, life-threatening neurodegenerative disease that affects 30–90% of diabetic patients worldwide. Multiple signalling pathways play an important role in the development and pathogenesis of diabetic neuropathy (Dewanjee et al. 2018). In this study, we used STZ to induce hyperglycaemia in rats. STZ can cause pancreatic selective β-cell toxin, and induce irreversible β-cell necrosis (RAKIETEN et al. 1963). Importantly, it was shown that diabetic rats induced with STZ at doses exceeding 40 mg/kg remained hyperglycaemic for 98 days (Dirice et al. 2009). And this study confirmed for the first time that veratramine has a good analgesic effect on DPN in rats, and using WB and co-IP technology, we examined the possible veratramine target SIGMAR1 and its downstream pathway, and present evidence for the regulation of SIGMAR1 by veratramine.

SIGMAR1 is localised in biofilms, including microsomes, mitochondria, nuclear membranes, and plasma membranes. From a functional perspective, SIGMAR1 physically interacts with various receptors, ion channels, or elements of their transduction machinery and acts as a regulator of their activity. In the plasma membrane, SIGMAR1 interacts with components of plasma membrane-bound signal transduction to regulate the activity of neurotransmitter receptors and ion channels, including K^+^ channels, Ca^2+^ channels, NMDARs and opioid receptors (Zamanillo et al. 2013). SIGMAR1 can also bind naturally occurring compounds, such as neurosteroids, pregnenolone, progesterone, dehydroepiandrosterone (DHEA), and their sulphates (Cobos et al. 2008; Su et al. 2010). SIGMAR1 has good compound binding characteristics, and it also plays an important role in neurotransmission and signal transduction.

SIGMAR1 is widely distributed in key areas of pain control, such as the superficial dorsal horn of the spinal cord, grey matter, and medulla (Alonso et al. 2000; Kitaichi et al. 2000). IHC results from our laboratory also confirmed that SIGMAR1 is widely expressed in the grey matter of the spinal cord. Recent studies have shown that direct activation of spinal SIGMAR1 in the pain environment of the spinal cord enhances NMDA-evoked spontaneous pain behaviour and produces nociceptive effects under certain altered or pathological conditions (Díaz et al. 2009; Romero et al. 2012). Additionally, it has been reported that the best-known protein target of SIGMAR1 is NMDAR (Sánchez-Fernández et al. 2017).

Clinical studies have shown that NMDAR antagonists can be used to treat neuropathic pain (Zhou et al. 2011; Welters et al. 2017). Activation of NMDARs leads to hyperreactivity of spinal nociceptive receptor neurons. Therefore, activation of NMDARs is able to promote the processing of pain in the spinal cord and lead to a sensation of pain and more severe pain (Inquimbert et al. 2018). Randomised double-blind clinical trials have further shown that NMDAR antagonists can reduce pain and pain sensitisation in patients with chronic neuropathic pain (Kalia et al. 2008). Therefore, we hypothesise that the high level of SIGMAR1 expression may have driven the activation of the NMDAR pathway (Rodríguez-Muñoz et al. 2021), which in turn mediates the response to chronic pain.

We found that Ca^2+^ homeostasis plays an important regulatory role between SIGMAR1 and NMDARs. Previous studies have shown that both neurons and glial cells experience metabolic stress and mitochondrial dysfunction in a rat model of STZ-induced diabetes, which leads to dysregulation of Ca^2+^ homeostasis, impaired Ca^2+^ buffering in mitochondria, and dysregulation of Ca^2+^ accumulation and Ca^2+^ signalling due to endoplasmic reticulum stress, which in turn promotes the development of DPN (Verkhratsky and Fernyhough 2014). However, the increase in intracellular Ca^2+^ also leads to the enhanced binding of Ca^2+^-calcium/calmodulin-dependent protein kinase II alpha (CaMKII) complexes to NMDARs. The latter event constitutes a Ca^2+^-dependent feedback mechanism that inhibits NMDARs, thereby preventing excess entry of Ca^2+^ into the cytoplasm.

In this complex process, SIGMAR1 plays a key role in the functional interaction between μ-opioid receptors and NMDA. SIGMAR1 is located in a complex formed by the μ-opioid receptors HINT1 and NMDAR. When NMDARs are active, the influx of Ca^2+^ induces SIGMAR1 binding to NMDARs, protecting the latter from the inhibitory effects of Ca^2+^-CaMKII (Rodríguez-Muñoz et al. 2015a, 2015b). Briefly, NMDAR activation promotes Ca^2+^ influx and SIGMAR1 interaction with NMDARs, which hinders the inhibitory effect of Ca^2+^-CaMKII on NMDARs. That is, after the maintenance effect of SIGMAR1 on the Ca^2+^-CaMKII-bound structure is diminished, CaMKII can easily interact with NMDARs and reduce their activity. Additionally, when NMDARs do not bind SIGMAR1, they are more susceptible to inhibition by Ca^2+^-CaMKII (Sánchez-Fernández et al. 2017).

With this evidence in mind, we examined the key action point of SIGMAR1 in pain. On the one hand, SIGMAR1 can bind to NMDARs and promote the activation of the NMDA pathway. On the other hand, veratramine can form a stable complex with Ca^2+^-CaMKII, which in turn prevents Ca^2+^-CaMKII from inhibiting the activity of NMDAR. In our study, we combined Co-IP experiments to study the protein binding level between SIGMAR1 and NMDAR and verified the protein binding level between NMDAR and CaMKII, and the results supported our hypothesis. The level of SIGMAR1 binding to NMDAR was enhanced in the model group, and the protein level between NMDAR and CaMKII was reduced, an effect that was significantly inhibited after veratramine treatment. This shows that the NMDAR pathway is likely to be activated in the DPN model group, while veratramine is likely to regulate NMDAR pathway levels through SIGMAR1, modifying NMDAR phosphorylation levels. Our results showed that the phosphorylation level of NMDAR at Ser896 was significantly inhibited. While some scholars have reported as early as 1997 that CaMKII inactivates NMDA channels by binding to NMDAR, PKA-mediated phosphorylation at Ser896 of NMDAR reduces the affinity of NMDAR for CaMKII (Hisatsune et al. 1997). This also supports the results of our Co-IP experiment.

In addition, pathologically, we observed that axonal degeneration occurred in the sciatic nerve of rats in the model group, accompanied by a decrease in the number of large fibres. However, large fibre degeneration is only one type of degeneration to occur in the pathogenesis of DPN, and it is only a small part (Gonçalves et al. 2018). The degeneration of small fibres is characterised by increased sensitivity to heat and cold. In this study, we observed that rats in the cold and heat DPN model group had increased sensitivity to cold and heat allodynia, while veratramine treatment resulted in decreased sensitivity to cold and heat allodynia. This shows that veratramine has a special effect on nerve fibre protection.

## Conclusions

In this study, we found that veratramine significantly alleviated mechanical pain and heat and cold pain induced by peripheral neuropathy in elderly rats with DPN, as well as alleviating the effect of diabetic nerve injury. We propose that veratramine exerts its effects in DPN by first weakening SIGMAR1-NMDAR pathway activation and inhibiting NMDAR Ser896 phosphorylation by inhibiting SIGMAR1 expression. Then, NMDAR phosphorylation activity decreases, thus, enhancing the affinity of NMDARs for CaMKII and increasing the probability of NMDAR inactivation. Finally, veratramine inhibits SIGMAR1 protein, thereby reducing Ca^2+^-CaMKII-stable complex formation, and more Ca^2+^-CaMKII inhibits the activity of NMDAR.

## Consent for publication

All of the authors have read and approved the final manuscript and agreed to publish.

## Data Availability

The datasets used in the current study are available from the corresponding author upon reasonable request.
